# A Rise in ATP, ROS, and Mitochondrial Content upon Glucose Withdrawal Correlates with a Dysregulated Mitochondria Turnover Mediated by the Activation of the Protein Deacetylase SIRT1

**DOI:** 10.3390/cells8010011

**Published:** 2018-12-27

**Authors:** Seon Beom Song, Eun Seong Hwang

**Affiliations:** Department of Life Science, University of Seoul, Dongdaemun-Gu, Seoul 02504, Korea; scitiger@naver.com

**Keywords:** ATP, mitochondria, glycolysis, glucose withdrawal, SIRT1, autophagy

## Abstract

Glucose withdrawal has been used as a model for the study of homeostatic defense mechanisms, especially for how cells cope with a shortage of nutrient supply by enhancing catabolism. However, detailed cellular responses to glucose withdrawal have been poorly studied, and are controversial. In this study, we determined how glucose withdrawal affects mitochondrial activity, and the quantity and the role of SIRT1 in these changes. The results of our study indicate a substantial increase in ATP production from mitochondria, through an elevation of mitochondrial biogenesis, mediated by SIRT1 activation that is driven by increased NAD^+^/NADH ratio. Moreover, mitochondria persisted in the cells as elongated forms, and apparently evaded mitophagic removal. This led to a steady increase in mitochondria content and the reactive oxygen species (ROS) generated from them, indicating failure in ATP and ROS homeostasis, due to a misbalance in SIRT1-mediated mitochondria turnover in conditions of glucose withdrawal. Our results suggest that SIRT1 activation alone cannot properly manage energy homeostasis under certain metabolic crisis conditions.

## 1. Introduction

Nutrient starvation is thought to impose a heavy impact on cell metabolism and viability, and therefore, it has been adopted as an important research model in studies to understand metabolic regulation and homeostatic defense mechanisms of cells. At early stages of nutrient starvation, cells tend to cope with a shortage of nutrient supply by enhancing catabolism. In particular, a decrease in adenosine triphosphate (ATP) generation and a concordant increase in ADP and AMP levels sends a catabolic cue, through the activation of AMP-activated protein kinase (AMPK) [1,2]. Activated AMPK drives a shift in cellular energy metabolism by inhibiting protein synthesis, and stimulating fatty acid oxidation and mitochondrial biogenesis, through the modulation of the activities of key enzymes involved in the processes [3,4,5]. AMPK also triggers autophagy, a representative cellular salvage pathway for recycling energy resources [4]. Therefore, AMPK activation and autophagy are considered to be hallmarks of the cellular response to energy deprivation conditions.

Glucose withdrawal has been used as one of the study models for nutrient starvation. However, the detailed cellular responses to glucose withdrawal are poorly understood, and even controversial. Cancer-derived cells, which produce ATP mainly through oxidative glycolysis, are expected to suffer from a depletion of ATP upon glucose withdrawal. Indeed, glucose-deprived cancer cells undergo cell death [6], which appears to be due to ATP depletion [7,8,9] or severe oxidative stress imposed by dysfunctional mitochondria activation [10,11,12,13,14]. Moreover, the effect of glucose withdrawal in normal cells is unclear; cell viability is not significantly affected in some cells [13,15], whereas apoptotic or non-apoptotic death has been observed in others [16]. The status of ATP level is also controversial. Studies have reported the activation of AMPK, due to an increase in the AMP/ATP ratio [17], or a substantial reduction in ATP level in various types of cells that are cultured in low or no glucose [16,18,19,20,21,22]. Unaltered or increased ATP levels was also reported in studies of glucose withdrawal in vitro and in animals [12,23]. In these cases, AMPK might be activated via redox-based signaling, such as a high level of reactive oxygen species (ROS) [24,25]. It is reasonably predicted, and has been shown that in conditions of glucose withdrawal, cells heavily rely on mitochondria for ATP production, and mitochondrial oxidative phosphorylation (OXPHOS) increases [26]. However, there are reports of contrary cases. Glucose deprivation in the retina in the absence of any other nutrients causes mitochondrial depolarization and an irreversible decrease in oxygen consumption rate, leading to cell death, which is prevented by supplementation with fuel molecules for oxidative phosphorylation [27]. Overall, how mitochondrial function is affected by glucose withdrawal has not been clearly resolved. Previous studies have proposed that Sirtuin 1 (SIRT1), an NAD^+^-dependent protein deacetylase, is activated through the AMPK-mediated regulation of NAD^+^ production, and plays a role in cell differentiation [22], and that SIRT1 acts as a link between the energetic stress response and metabolism in calorie restriction [28]. However, little is known about the role of SIRT1 in mitochondrial status during glucose withdrawal. Recent studies have demonstrated that SIRT1 activity is critically associated with mitochondrial quality, as well as quantity. SIRT1, either alone or in collaboration with AMPK, activates peroxisome proliferator-activated receptor gamma coactivator-1α (PGC-1α), a master regulator of the expression of mitochondria proteins, and thereby upregulates mitochondria biogenesis [29]. In addition, SIRT1 and AMPK promote autophagy via deacetylation-mediated activation of autophagy-related gene (ATG) proteins, which function in autophagosome formation [30,31]. Therefore, SIRT1 activation facilitates mitochondria turnover, and thereby can promote mitochondria quality in proliferating cells, which is of upmost importance for cellular health and longevity [32], as indicated by the loss of mitochondrial turnover and the accumulation of dysfunctional mitochondria and ROS stress in senescent and aging cells [33]. In cells under glucose deprivation, the reduction of NAD^+^ to NADH would decrease, and SIRT1 activity is expected to increase, as demonstrated under similar conditions of calorie restriction [34]. Based on these, it can be postulated that cells under glucose withdrawal can produce ATP efficiently from mitochondria without experiencing a high level of ROS production. However, this is apparently contradictory to the changes in ROS level observed in many studies on glucose withdrawal.

In this study, we determined how the activities of SIRT1 change in human cells upon glucose withdrawal and affect mitochondrial status. The results of our study indicate that a substantial increase in ATP production occurs through SIRT1-mediated elevation of mitochondrial biogenesis, which are not matched by mitochondrial turnover, and therefore leads to a continuous increase in mitochondria content and ROS generation. These suggest that SIRT1 activation alone cannot properly manage energy homeostasis and oxidative balance under certain metabolic crisis conditions.

## 2. Materials and Methods

### 2.1. Cell Culture and Chemical Treatments

Normal human fibroblasts isolated from healthy newborn foreskins and provided by Dr. Jin-Ho Chung (Seoul National University, Korea) were cultured in Dulbecco’s modified Eagle’s medium (DMEM) containing 1 g/L glucose (12-707F, Lonza, Walkersville, MD, USA) supplemented with 10% fetal bovine serum (FBS) (S-FBS-US-015, Serana, Bunbury, WA, Australia) and glutamine (25030-081, Gibco, Waltham, MA, USA). Human cancer lines MCF-7 and HCT116 were maintained in Dulbecco’s modified Eagle’s medium (DMEM) containing 4.5 g/L glucose (12-107F, Lonza, Walkersville, MD, USA) supplemented with 10% fetal bovine serum (FBS) (S-FBS-US-015, Serana, Bunbury, WA, Australia) at 5% CO_2_ and 37 °C. For glucose withdrawal, DMEM free of glucose (LM001-79, Welgene, Daegu, Korea) was used. Cells were treated with 5 mM *N*-acetylcysteine (NAC) (A7250, Sigma-Aldrich, St. Louis, MO, USA), 10 μM EX527 (E7034, Sigma-Aldrich), 2 μM compound C (ab120843, Abcam, Cambridge, UK), 5 mM 3-methyladenine (M9281, Sigma-Aldrich), and 250 nM Torin1 (orb146133, Biorbyt, Cambridge, UK). Other chemicals were purchased from Sigma-Aldrich unless stated otherwise.

### 2.2. ATP Measurement

Equal numbers of cells (typically in the range of 1–3 × 10^5^) were lysed to determine the level of cellular ATP, using an ATP detection kit (LT07-221, ViaLight Plus kit, Lonza, Basel, Switzerland) according to the manufacturer’s protocol. Chemiluminescence was read in an FL × 800 microplate fluorescence reader (Biotek, Winooski, VT, USA). ATP production independent of oxidative phosphorylation (OXPHOS) was determined using cells treated with either 2 μM oligomycin A (75351) or a combination of 1 μM rotenone (R8875) and 1 μM antimycin A (A8674) for 1 h before collection. Both treatments gave rise to quite similar results, as seen in Figure S1A,B. ATP production by oxidative phosphorylation was estimated by subtracting ATP produced in rotenone and antimycin A–treated cells from total cellular ATP.

### 2.3. Measurements of OCR and ECAR

To measure mitochondrial respiratory activity, the cellular oxygen consumption rate (OCR) was detected using a Seahorse XF24 (Seahorse Bioscience, Inc., North Billerica, MA, USA), following the manufacturer’s instructions. Briefly, cells were seeded onto the XF24 culture plate and incubated for one day in a 5% CO_2_ incubator at 37 °C. The following day, cells were pre-incubated for 1 h in XF base medium (102353-100, Seahorse Bioscience) containing 1 mM sodium pyruvate (P5280, Sigma-Aldrich), 4 mM glutamine (25030-081, Gibco, Waltham, MA, USA), and 1% FBS at 37 °C without glucose. The XF24 culture plate was mounted in an analyzer, and OCR was measured and represented as pmoles/min. Extracellular acidification rate (ECAR) was measured simultaneously and represented as mpH/min. For the measurement of OCR in cells treated with 2-deoxy-D glucose (2-DG), seeded cells were pre-incubated for 1 h in XF base medium with 10 mM 2-DG (D6134, Sigma-Aldrich) before the measurement of OCR. All experiments were performed at least four times, and OCR was expressed as the fold-change relative to the values obtained from untreated control cells.

### 2.4. siRNA Transfection

Cells were transfected with small interfering RNA (siRNA) corresponding to human SIRT1 or Mfn1, or control RNA using Lipofectamine RNAiMAX (13778-150, Thermo Scientific, Waltham, MA, USA) according to the manufacturer’s protocol. siSIRT1 (pre-designed, 23411, Bioneer, Daejun, Korea); siMfn1 (pre-designed, 55669, Bioneer, Daejun, Korea) or control RNA (siCtrl; CCUACGCCACCAAUUUCGU (dTdT)) (Bioneer, Daejun, Korea) were used.

### 2.5. Western Blot Analysis

Protein extraction was performed with RIPA buffer (50 mM Tris-HCL (pH 7.5), 150 mM NaCl, 1% Nonidet P-40, 0.5% sodium deoxycholate, 0.1% SDS) supplemented with NaF, NaVO_4_, and protease inhibitor cocktail (P2714). Proteins were separated by sodium dodecyl sulfate-polyacrylamide gel electrophoresis (SDS-PAGE), transferred to a nitrocellulose membrane, and blotted with antibodies against AMPKα phosphorylated at Thr172 (#2535), AMPKα (#2603), acetylated-p53 (#2525), LC3 (#2775), phospho-Drp1 (#4867) (all from Cell Signaling Technology, Beverly, MA, USA); Erk (SC-93), p53 (SC-126), GAPDH (SC-25778), PGC1α (SC-13067), and Mfn1 (SC-166644) (all from Santa Cruz Biotechnology, Dallas, TX, USA); β-actin (A5441, Sigma-Aldrich); Drp1 (611112, BD, Franklin Lakes, NJ, USA); and OXPHOS (MS601, Mitosciences, Eugene, OR, USA). Proteins were visualized by using horseradish peroxidase-conjugated secondary antibodies and Supersignal West Femto substrate (34095, Thermo Scientific).

### 2.6. Confocal Microscopy

Cells grown on coverslips were fixed in 3.7% paraformaldehyde in phosphate-buffered saline (PBS) for 20 min, permeabilized with 0.1% Triton X-100 in PBS for 15 min, blocked with 10% fetal bovine serum (FBS) in PBS for 2 h, and incubated with primary antibodies overnight. For the detection of mitochondria, antibodies against OXPHOS (MS601, Mitosciences, Eugene, OR, USA) and Tom20 (SC-11415, Santa Cruz Biotechnology, Dallas, TX, USA) were used. Autophagosomes were stained with antibody against LC3 (#2775, Cell Signaling Technology, Beverly, MA, USA). After incubation with primary antibody, the cells were washed and incubated with Alexa Fluor 488-conjugated anti-mouse, Alexa Fluor 488-conjugated anti-rabbit, or Alexa Fluor 546-conjugated anti-rabbit secondary antibodies (all from Thermo Fisher, Waltham, MA, United States) for 2 h, and visualized under a confocal microscope (LSM 510, Carl Zeiss, Thornwood, NY, USA). Mitochondrial length was measured using ImageJ analysis software (v1.52e, National Institutes of Health, Bethesda, MD, USA).

### 2.7. Flow Cytometry

For the measurement of mitochondria content or ROS levels, the cells were stained for 30 min with 50 nM nonyl acridine orange (NAO) (A1372, Thermo Fisher, Waltham, MA, United States), 30 nM MitoTrackerGreen (MTG, M7514, Thermo Fisher, Waltham, MA, United States), 50 nM MitotrackerDeepRed (MDR, M22426, Thermo Fisher, Waltham, MA, United States), 50 nM dihydroethidium (DHE, D1168, Thermo Fisher, Waltham, MA, United States), 50 nM MitoSOX (M36008, Thermo Fisher, Waltham, MA, United States), 50 nM 2’,7’–dichlorofluorescin diacetate (DCFDA, C6837, Thermo Fisher, Waltham, MA, United States), or 50 nM dihydrorhodamine 123 (DHR123, D23806, Thermo Fisher, Waltham, MA, United States), as indicated. After washing in PBS, cells were analyzed by flow cytometry using a FACS Canto II (BD Biosciences, Franklin Lakes, NJ, USA). For measurement of mitochondrial membrane potential (Δψ_m_), cells were treated with 0.3 μg/mL JC-1 (T3168, Thermo Fisher, Waltham, MA, United States) and subjected to flow cytometry. Emissions at 539 nm (FL-1) and at 585 nm (FL-2) were recorded, and the FL2/FL1 ratio of individual cells was calculated using WEASEL software v3.2.1 (Chromocyte, Sheffield, UK).

### 2.8. Quantitative PCR

Total RNA was isolated using TRIzol reagent (Thermo Fisher, Waltham, MA, United States) and converted to complementary DNA (cDNA) using a reverse transcription kit (ReverTra Ace qPCR RT kit, FSQ-101, TOYOBO, Osaka, Japan). cDNA was diluted with autoclaved water and mixed with SYBR Green for quantitative polymerase chain reaction (qPCR) (CFX connect, BioRad, Hercules, CA, USA). The sequences of the primers used are listed in the Supplementary Data (Figure S7). To determine the relative copy number of the mitochondrial DNA (mtDNA), total cellular DNA was isolated using an AccuPrep Genomic DNA extraction kit (Bioneer, Daejeon, Korea) following the manufacturer’s protocol. Isolated DNA was amplified using primers for mitochondrial transfer DNA (tRNA)-Leu or nuclear B2-microglobulin whose sequences are listed as Supplementary Data (Figure S7).

### 2.9. Measurement of NAD^+^/NADH

The ratio of NAD^+^/NADH was determined by a lactate/pyruvate oxidase assay, as described in [35]. Briefly, for the lactate oxidase assay, trypsinized cells were lysed with 100 μL ice-cold 1 M HClO_4_, followed by incubation for 15 min on ice. Acid extracts were neutralized by the addition of 2 M KOH and 0.2 M K_3_PO_4_ (pH 7.5), and centrifuged for 3 min at 13,000× *g*. The cell lysate was mixed with 196 μL of assay buffer (0.1 M citrate, 1 mg/mL bovine serum albumin (BSA), 0.1% CaCl_2_, 0.02% NaN_3_, adjusted to pH 6.5 with 1 M Na_2_HPO_4_), 1 μL of 2 mU/μL lactate oxidase stock (lactate oxidase enzymes (Sigma-Aldrich, St. Louis, MO, USA) dissolved in enzyme dilution buffer (10 mM KH_2_PO_4_, 10 μM flavin adenine dinucleotide (FAD), adjusted to pH 7.0 with KOH), 1 μL of 0.5 U/μL peroxidase stock (peroxidase (Sigma, St. Louis, MO, USA) dissolved in distilled water), and 2 μL of 5 mM Amplex UltraRed stock (Thermo Fisher, Waltham, MA, United States). Assay mixtures were incubated for 30 min at 37 °C, and fluorescence was read at an excitation/emission of 535/590 nm. For the pyruvate oxidase assay, cells were lysed as described above, and mixed with 196 μL of assay buffer (50 mM KH_2_PO_4_, 1 mg/mL BSA, 0.2 mM triphenylphosphine, 10 μM FAD, 0.97 mM EDTA, 9.8 mM MgCl_2_, 0.02% NaN_3_, adjusted to pH 6.5 with 1 M NaOH), 1 μL of 2 mU/μL lactate oxidase stock (pyruvate oxidase dissolved in buffer (10 mM KH2PO4, 10 μM FAD, adjusted to pH 7.0 with KOH), 1 μL of 0.5 U/μL peroxidase stock, and 2 μL of 5 mM Amplex UltraRed stock. Assay mixtures were incubated for 60 min at 37 °C, and fluorescence was read at an excitation/emission of 535/590 nm.

### 2.10. Statistical Analysis

In all panels, quantifications were performed with two or three independent measurements of samples from two different experiments, and the mean ± S.E.M values were presented. Intergroup comparison of the mean values was performed by one-way analysis of variance (ANOVA) using InStat v3.06 (GraphPad Software Inc., San Diego, CA, USA). Significant differences are indicated as *(*p* > 0.1) and **(*p* < 0.01).

## 3. Results

### 3.1. Cellular ATP Level is Enhanced Upon Glucose Withdrawal Through Increased Mitochondrial ATP Production

**Figure 1:**
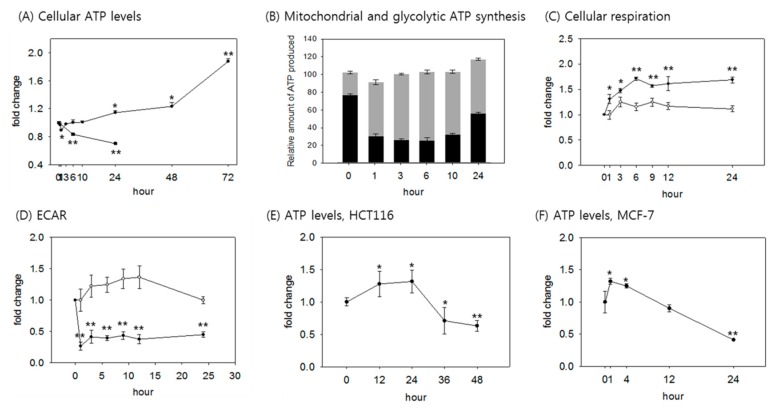
Increase in cellular ATP level majorly through enhanced mitochondrial ATP production in glucose-deprived cells. (**A**) Human fibroblasts were cultivated in glucose-free (-●-) or serum-free (-■-) medium for the indicated time, and harvested for ATP measurement. For determination of ATP levels, at least two biological repeats were carried out; (**B**) changes in glycolytic and mitochondrial ATP productions were measured at the indicated time points. For the inhibition of OXPHOS, cells were treated with 1 μM antimycin A and 1 μM rotenone for the last hour of glucose withdrawal. Glycolytic ATP was determined by the measurement of ATP level in cells treated with OXPHOS inhibitor. Mitochondrial ATP production was calculated by the subtraction of glycolytic ATP from total ATP level. Changes in the total ATP level and the portions of the glycolytic (black) and mitochondrial (grey) ATP production are plotted with total ATP level, with 0 time-point cells presented as 100%; (**C**,**D**) fibroblasts were cultivated in medium lacking glucose and incubated on an XF24 culture plate for 24 h. Oxygen consumption and extracellular acidification were measured using an XF24 analyzer. (-○-) and (-●-) show the values in cells incubated with normal medium and glucose-free medium, respectively. Values of total oxygen consumption or extracellular acidification rate were measured three times. (**E**,**F**) HCT116 or MCF7 cells were incubated without glucose for the indicated times, and cellular total ATP was measured. ATP levels were determined in at least two biological repeats. Values are presented as mean ± s.d. * *p* < 0.05; ** *p* < 0.01 by ANOVA.

We first determined how cellular, mitochondrial, and glycolytic levels of ATP production change upon glucose withdrawal. Human fibroblasts that had been maintained in DMEM containing 5.5 mM glucose were cultivated in media lacking glucose, and changes in ATP level were followed for three days. The ATP level acutely decreased immediately after glucose withdrawal, but quickly returned to the original level and then further increased, reaching 1.2–1.3-fold elevation in 24 h and near 2-fold increase in 72 h (Figure 1A). An increase in ATP level was also observed in other fibroblast lines tested, albeit with a variation in kinetics (Supplemental Figure S1A,B). This pattern contrasts sharply with that of cells undergoing quiescence induced by serum starvation, in which the cellular ATP level decreased continuously for 24 h (Figure 1A). This increase in ATP level does not appear to be caused by a decrease in consumption. The degree of decrease in ATP level when ATP production was completely blocked for 2 h (by combined treatment with 2-deoxyglucose (2-DG) and oligomycin), as an indication of ATP consumption during the 2 h, was not different between glucose-fed and glucose-deprived cells (Figure S1D). Furthermore, the absence of glucose in the culture medium did not cause a change in the level of population growth, at least for the first 24 h (Figure S1E). Therefore, the increase in the ATP level is attributed solely to enhanced ATP production. The level of mitochondrial ATP synthesis was determined by the decrease in ATP level caused by treatment with oligomycin A, which inhibits ATP synthase (or combined treatment of rotenone and antimycin A, which inhibit complex I and III, respectively), and abolishes OXPHOS-mediated ATP production [36,37]. In the glucose-fed condition, the tested fibroblasts produced nearly 75% of total ATP through glycolysis, and 25% via OXPHOS (Figure 1B). In contrast, upon glucose withdrawal, mitochondrial ATP production increased rapidly, while glycolytic production decreased, and the ratio was reversed as early as in 1 h. A parallel increase in total cellular oxygen consumption supports increased OXPHOS (Figure 1C). A decrease in glycolytic flux was also demonstrated by a rapid decrease in the rate of extracellular acidification (ECAR) (Figure 1D). Interestingly, glycolytic ATP synthesis decreased, but was not abolished, and increased after 10 h of glucose withdrawal, approaching 2/3 levels of the fed cells by 24 h (Figure 1B). Overall, by 24 h, the cells produced an elevated level of ATP, through a large increase in OXPHOS. After 24 h, the cells stopped dividing (Figure S1E), and therefore, the rapid increase in ATP level in 48 h and later might be attributed in part to the lower ATP consumption.

Cancer cells are less dependent on OXPHOS [38,39], and are therefore expected to respond differently to ATP production upon glucose withdrawal. The ATP level was elevated as an initial response in the two cancer cell lines tested, HCT116 and MCF7, although the elevation was not sustained for long, and the kinetics of the change were different between the cell lines (Figure 1E,F). This result suggests that OXPHOS is mobilized to a certain extent to produce ATP upon glucose withdrawal in cancer cells as well.

### 3.2. AMPK is Activated by Increased ROS Level 

**Figure 2:**
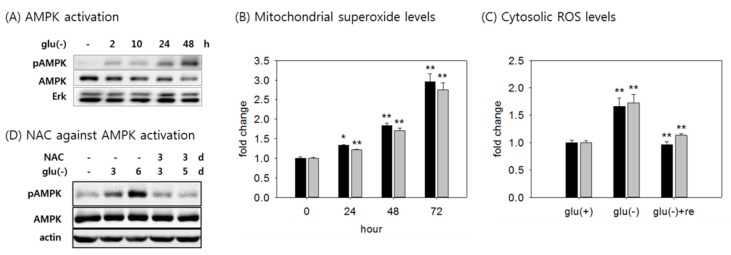
Activation of AMPK by increased ROS level. (**A**) Cells were incubated in glucose-free medium for the indicated time, and the activity of AMPK was detected by immunoblot analysis with antibodies against phospho-AMPK and AMPK; (**B**) for the measurements of mitochondrial and cytosolic superoxide, cells were glucose-deprived for the indicated time, stained with mitoSOX (black bar) and dihydroethidium (DHE) fluorescent dyes (grey bar), and more than 1 × 10^4^ cells were analyzed by flow cytometry. The levels of signal at each time point was determined in at least three biological repeats; (**C**) mitochondrial and cytosolic ROS were detected using DHR123 (black bar) and DCF (grey bar) fluorescent dyes, respectively. To measure the effect of glucose replenishment on the cellular ROS levels, cells were cultivated in glucose-free medium for three days, and then supplied with glucose for one day; (**D**) Cells were incubated in glucose-free medium with 5 mM N-acetylcysteine (NAC) for the indicated time period. Cells of lane 5 were treated with NAC for the last three days of withdrawal. Cells were lysed and subjected to immunoblotting with antibodies against phospho-AMPK and AMPK. Values are presented as mean ± s.d. * *p* <0.05; ** *p* < 0.01 by ANOVA.

A reported change that accompanies glucose withdrawal is the activation of AMPK. Indeed, in both the fibroblasts and the cancer cells tested, activating the phosphorylation of AMPK increased gradually during glucose withdrawal (Figure 2A and Figure S2A). AMPK activation in cells undergoing glucose withdrawal has been attributed to a decrease in cellular ATP level [17], which was apparently not the case in this study. AMPK activation can also be driven by an increase in ROS [16,40]. Indeed, the levels of both mitochondrial and cytosolic superoxide substantially increased in the glucose-deprived cells, and this change occurred with kinetics that were comparable to the change in AMPK phosphorylation (Figure 2B). Accordingly, the levels of hydroxyl radicals in both mitochondria and cytosol increased upon glucose withdrawal (Figure 2C). ROS levels were reduced to a background level upon glucose re-feeding (Figure 2C). In the tested cancer cells, the mitochondrial ROS level also increased rapidly (Figure S2B). The possibility of the ROS-mediated induction of AMPK is strongly supported by the results of treatment with *n*-acetyl cysteine (NAC), which abolished AMPK activation in the starved cells (Figure 2D, lanes 4 and 5).

### 3.3. An Increase in Mitochondrial ATP Production is Mediated by Activated SIRT1 and AMPK

**Figure 3:**
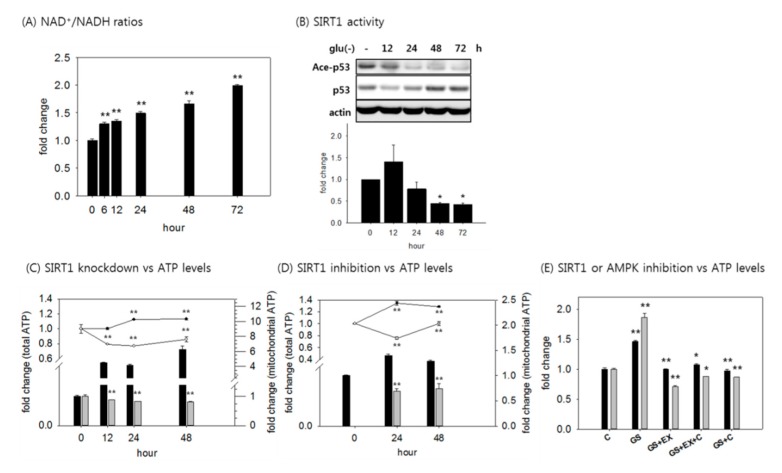
An increase in mitochondrial ATP production was mediated by the activation of SIRT1 and AMPK. (**A**) The cellular NAD+/NADH ratio was determined by measuring NAD+ and NADH levels at the indicated times; (**B**) Cells were deprived for glucose for 72 h, and then subjected to immunoblotting with antibodies against acetylated-p53 and p53. The activity of SIRT1 was calculated and represented as the ratio of acetylated-p53 to total p53 protein level; (**C**) Total ATP and mitochondrial ATP production levels. Cells were treated with nonspecific RNA (siCtrl) or siSIRT1 RNA, and then incubated in the presence or absence of glucose for the indicated time period. To determine mitochondrial ATP production, cells were treated with both 1 μM antimycin A and 1 μM rotenone for the last hour. The glycolytic ATP level was subtracted from the total ATP level. The line plot shows total ATP level in the control (-●-) or SIRT1-knocked down (-○-) cells, and the bar chart shows the mitochondrial ATP production level in the control (black bar) or SIRT1-knocked down (grey bar) cells; (**D**) Total ATP and mitochondrial ATP production were plotted as shown in (**C**). Cells were incubated in glucose-free medium with or without 10 μM EX527 for 48 h, and lysed for the measurement of ATP levels; (**E**) Cells were incubated in glucose-free medium for three days, and treated with 10 μM EX527 and 2 μM compound C for the last 12 h. Black bar shows the total ATP level, and grey bar shows the mitochondrial ATP level. Averages of values from at least two biological repeats are plotted. Values are presented as mean ± s.d. * *p* < 0.05, ** *p* < 0.01 by ANOVA.

In the situation of a low level of glycolysis and increased OXPHOS, the reduction of NAD^+^ would be limited, and cells would maintain a high NAD^+^/NADH ratio. This was indeed the case in the tested fibroblasts; the NAD^+^/NADH ratio gradually increased, reaching near 1.5-fold elevation in 24 h, and further increasing at later time points of glucose withdrawal (Figure 3A). This is a condition that is favorable for SIRT1 activation, and increased SIRT1 activity was evidenced by a decrease in the acetylation level of p53, a substrate of SIRT1 [41] (Figure 3B). SIRT1 activation promotes mitochondria quality by facilitating mitochondria biogenesis and mitochondrial autophagy (mitophagy) [42,43]. We therefore examined whether SIRT1 activation contributed to the increase in mitochondrial ATP production. In cells with suppressed SIRT1 expression or activity (through treatment with siRNA or EX527, an inhibitor of SIRT1), ATP level in the starved cells did not increase, or slightly decreased (Figure 3C,D). Mitochondrial ATP production also decreased, and never increased in the SIRT1-suppressed cells (Figure 3C,D). These data strongly suggest that the increase in mitochondrial ATP production might be largely attributed to SIRT1 activity, which was increased through the elevation of NAD^+^/NADH upon glucose withdrawal. Next, we determined the contribution of activated AMPK to the changes in ATP level. In cells treated with compound C, an inhibitor of AMPK, the cellular and mitochondrial ATP levels never increased, similar to cells treated with EX527 (Figure 3E). Moreover, co-treatment with compound C and Ex527 did not affect the change in cellular and mitochondrial ATP levels induced by treatment with either inhibitor alone (Figure 3E). Together, these findings indicate that SIRT1 and AMPK caused an increase in ATP production through a common pathway.

### 3.4. SIRT1 Activation is Responsible for the Increased ATP Level in Glucose-Deprived Cells Through Enhancing Mitochondrial Biogenesis and Glycolysis

**Figure 4:**
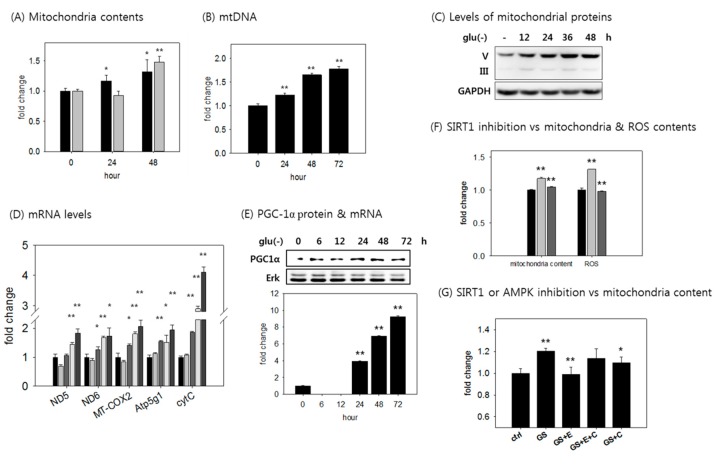
Increased mitochondrial content resulting from elevated mitochondria biogenesis through SIRT1 activation. (**A**) Fibroblasts were incubated in glucose-free medium for the indicated time period, and analyzed by flow cytometry. Mitochondrial content was detected by staining with MTG (black bar) or NAO (grey bar); (**B**) mitochondrial DNA copy number was determined by qPCR. Genomic DNA was isolated, and the relative mtDNA amount was measured by qPCR normalized against the amount of nuclear DNA; (**C**) mitochondrial electron transport chain complex III or V was detected in cells incubated in glucose-free medium by immunoblotting with OXPHOS cocktail antibody; (**D**) transcription of three genes encoded by the mitochondrial genome, NADH dehydrogenase, subunits 5 and 6 (ND5, ND6), and cytochrome c oxidase subunit II (MT-COX2), and two genes encoded by the nuclear genome, ATP synthase lipid-binding protein (Atp5g1) and cytochrome c, were measured by qRT-PCR. Cells were incubated without glucose for four days, and then lysed. The means of three biological repeats of the control, and 24, 48, 72, and 96 h deprivation are indicated as the bars in order; (**E**) PGC-1α protein expression was detected by immunoblot analysis and confirmed by qRT-PCR; (**F**) cells were glucose deprived for three days, and mitochondrial content or mitochondrial superoxide was determined by staining with MDR or mitoSOX and analysis by flow cytometry. Black bar; control, light grey bar; glucose withdrawal, dark grey bar; glucose withdrawal and 10 μM EX527 treatment; (**G**) cells were glucose deprived for three days and treated with 10 μM EX527 (GS + E) or 2 μM compound C (GS + C), or both (GS + E + C) for the last 12 h. Mitochondrial content was determined by staining with MDR, and analyzed by flow cytometry. All results from flow cytometry are the means of at least two biological repeats of more than 1 × 10^4^ cells. Values are presented as mean ± s.d. * *p* < 0.05, ** *p* < 0.01 by ANOVA.

We next investigated whether mitochondria biogenesis and mitophagy are actively ongoing in glucose-deprived fibroblasts. One prominent change in the starved cells was the increase in mitochondrial content, which was quite dramatic, as demonstrated by the increased fluorescence of mitochondria-trophic dyes (Figure 4A) and the levels of mitochondrial DNA and proteins (ATP5A of complex V and UQCRC2 of complex III) (Figure 4B,C). The mitochondrial content also increased in HCT116 and MCF7 cells (Figure 2C,D). This increase in mitochondrial content appeared to be attributed at least in part to an increase in mitochondria biogenesis. mRNA levels of five different mitochondrial proteins were all elevated throughout the 4-day time course of glucose withdrawal (Figure 4D), and this change was accompanied by an increase in the expression of PGC-1α, a master regulator of mitochondrial biogenesis [44]. Levels of PGC-1α mRNA and protein were both substantially elevated in the glucose-deprived cells (Figure 4E). *PGC-1α* expression is subject to auto-regulation in collaboration with SIRT1, which activates PGC-1α through deacetylation [45,46]. Importantly, the increase in mitochondrial content was attenuated upon treatment with EX527 (Figure 4F), suggesting that the increased mitochondria content is indeed attributed to SIRT1 activation-mediated biogenesis. Treatment with compound C alone or with EX527 also attenuated the increase in mitochondrial content, again suggesting that SIRT1 and AMPK function in a common pathway (Figure 4G). Intriguingly, the treatment of compound C appeared to lower the effect of EX527. This may be insignificant. However, the lower effect of compound C might actually be driven by its effect in blocking AMPK-induced autophagy [47,48,49]. Meanwhile, activated PGC-1α also induces gluconeogenesis by transcriptionally activating the genes encoding key gluconeogenic enzymes [50]. The increased glycolytic ATP production that occurred after 10 h of glucose withdrawal (Figure 1B) may be caused by PGC-1α activation. Therefore, SIRT1 activation might contribute to the high ATP level in glucose-deprived cells by promoting both mitochondrial biogenesis and gluconeogenesis.

### 3.5. Autophagy is Activated, but Mitophagy is Attenuated in Glucose-Deprived Cells.

**Figure 5:**
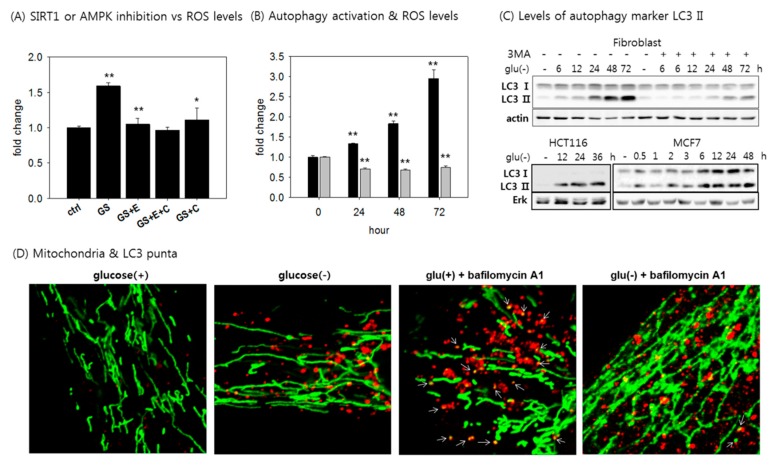
Activation of autophagy and the attenuation of mitophagy in glucose-deprived cells. (**A**) Cells were glucose deprived for three days in the presence or absence of 10 μM EX527 (GS + E) or 2 μM compound C (GS + C), or both (GS + E + C), stained with mitoSOX, and subjected to flow cytometric analysis; (**B**) cells were incubated in glucose-free medium (black bar), or treated with 250 nM torin1 (grey bar) for the indicated time period. Mitochondrial superoxide was stained with mitoSOX and detected by flow cytometry; (**C**) fibroblast, HCT116, or MCF7 cells were glucose-deprived in the presence or absence of 5 mM 3-MA for the indicated time points, and subjected to immunoblotting for LC3 protein; (**D**) cells were glucose-deprived for 12 h and treated with 200 nM bafilomycin A1 for 12 h, which has been shown to block mitophagy [52]. After fixing, mitochondria (green) and autophagosomes (red) were visualized with confocal microscopy using mouse anti-OXPHOS antibody and rabbit anti-LC3 antibody, respectively. All flow cytometry was carried out in, at least two biological repeats. All scale bars in microscopy indicate 5 μm. Values are presented as mean ± s.d.; * *p* < 0.05, ** *p* < 0.01 by ANOVA.

Next, we examined the status of mitophagy. SIRT1 and AMPK are key factors that are critically involved in autophagy activation; the former deacetylates Atg molecules [30], and the latter has been shown to modulate factors that control autophagy initiation, such as TORC1 and ULK1 [51]. Both SIRT1 and AMPK are activated upon glucose withdrawal, possibly to help cells to recycle resources and to enhance mitochondrial quality. However, the elevated ROS generation upon glucose withdrawal and its attenuation by the activation of SIRT1 or AMPK (Figure 5A) do not fit the hypothesis of increased mitochondrial quality through mitophagy activation. Autophagy activation helps to reduce ROS levels, as seen in the case of Torin 1 treatment (Figure 5B, grey bars). Furthermore, in the glucose-deprived cells, mitochondria content was not stable, but increased continuously. These findings together suggest a failure in removing mitochondria. In glucose-deprived fibroblasts and cancer cells, autophagy appeared to be activated, as evidenced by an increase in the LC3-type II molecule, which represents autophagosome formation, and LC3-positive puncta, which represent autophagosomes) (Figure 5C,D). However, mitochondria persisted in lengthy structures that are not suitable for mitophagy (Figure 5D). When cells with actively ongoing mitophagy are visualized, a population of mitochondria are seen as puncta, and some of them co-localize with autophagosomes [52] as shown in Figure 5D (glucose(+), bafilomycin A1). However, only a few LC3 puncta colocalized with mitochondria (Figure 5D (glucose (−), bafilomycin A1)), indicating a failure in the autophagasomal enclosure of mitochondria. This would lead to deficient mitochondrial turnover, which might contribute to the increase in mitochondrial content and ROS level in the glucose-deprived cells.

### 3.6. Mitochondria Elongate and Become Resistant to Mitophagy under Glucose Withdrawal

**Figure 6:**
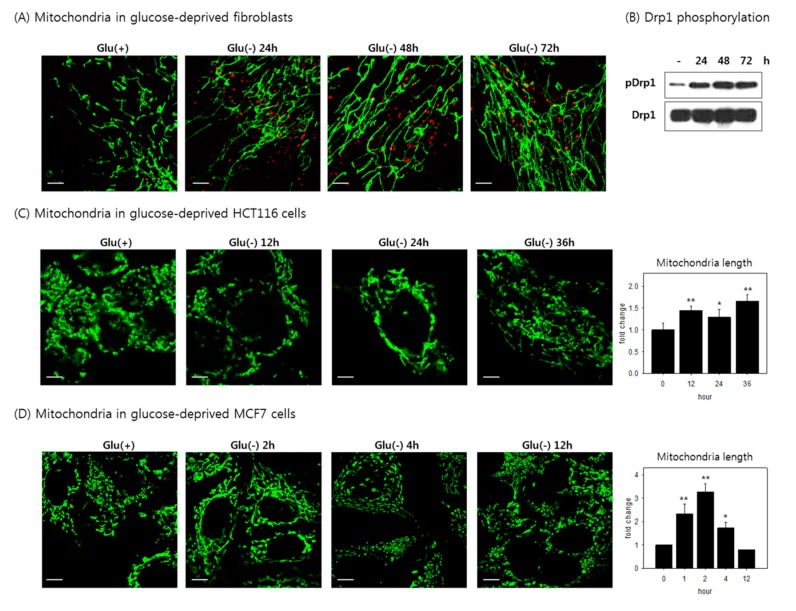
Elongation of mitochondria. (**A**) Fibroblasts cultured on a coverslip were deprived for the indicated time period and visualized by confocal microscopy. Mitochondria and autophagosomes were immunostained with antibodies against OXPHOS and LC3, respectively; (**B**) fibroblasts were glucose deprived for 1, 2, or 3 days, and subjected to immunoblot analysis with antibodies against Ser637 phospho-Drp1 or Drp1; (**C**,**D**) HCT116 or MCF7 cells were incubated in glucose-free medium for 36 h or 12 h. Mitochondria were immunostained with Tom20 antibody and observed by confocal microscopy. Lengths of mitochondria were measured and plotted by ImageJ analysis software. Lengths of mitochondria was measured in more than 20 cells and plotted by ImageJ analysis software. All scale bars in microscopy indicate 5 μm. Values are presented as mean ± s.d.; * *p* < 0.05, ** *p* < 0.01 by ANOVA.

In mitophagy, mitochondrial fission accompanies autophagy activation to facilitate the autophagosomal enclosure of target mitochondria [53]. Meanwhile, mitochondria have been proposed to form filamentous or tubular structures to avoid autophagosomal degradation during nutrient starvation [54]. Mitochondria in the glucose-deprived cells as well, became dramatically elongated, and were visualized as lengthy filaments (Figure 6A and Figure S3A). Mitochondrial length increased during the first 24 h, and the elongation persisted as glucose withdrawal continued. Together with this elongation, Drp1 phosphorylation at serine 637, a modification that negatively regulates the fission activity of Drp1 [55,56], increased substantially (Figure 6B), indicating that the mitochondrial elongation might be caused by Drp1 inactivation in the glucose-deprived fibroblasts. Drp1 phosphorylation and activity were shown to be regulated by protein kinase A (PKA) [57], whose activity is altered by energy stress [58]. Mitochondrial elongation was also observed in the cancer cells tested (Figure 6C,D). Interestingly, the period when mitochondria are elongated appeared to correlate with the duration of high cellular ATP level; in HCT116 cells, this period lasted for 36 h and thereafter declined, whereas in MCF7 cells, it increased rapidly and declined rather quickly. Overall, we hypothesized that the elongation of mitochondria rendered them resistant to mitophagy, as previously reported [54], resulting in an increased content of mitochondria. These changes in mitochondrial structure and quantity likely contributed to the elevation of ATP production and ROS generation in the glucose-deprived cells.

### 3.7. The Increase in Mitochondrial ATP Synthesis upon Glucose Withdrawal is Attributed to Increase in the Content of Mitochondria 

**Figure 7:**
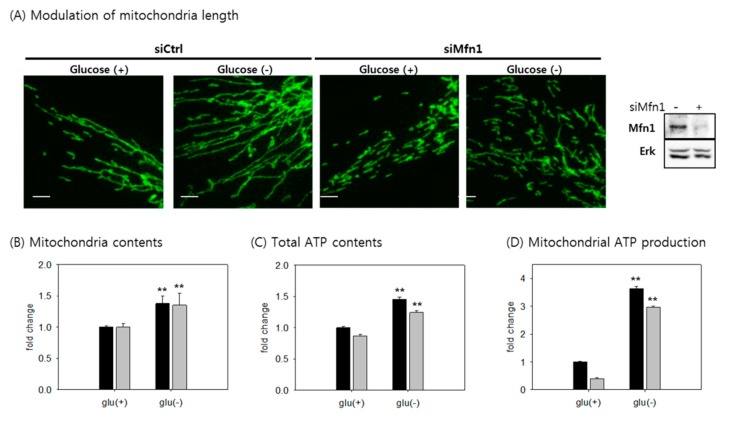
The increase in mitochondrial ATP production involves the increased content of mitochondria. (**A**) Cells transfected with siRNA targeting Mfn1 were incubated in medium lacking glucose for 72 h. Mitochondria were immunostained with anti-Tom20 antibody and visualized. The Mfn1 blot (right) shows decreased Mfn1 protein level in cells treated with siMfn1 RNA. All scale bars in microscopy indicate 5 μm; (**B**) cells were glucose deprived for three days, and mitochondrial content in cells treated with siCtrl (black bar) or siMfn1 (grey bar) was measured by staining with NAO and flow cytometric analysis. All flow cytometry was carried out in, at least two biological repeats; (**C**,**D**) total ATP level, or the amount of ATP produced by oxidative phosphorylation in cells treated with siCtrl (black bar) or siMfn1 (grey bar). Cells were glucose deprived for three days. Values are presented as mean ± s.d.; * *p* < 0.05, ** *p* < 0.01 by ANOVA.

Mitochondria fusion has been proposed as a cellular mechanism to increase ATP production [59,60]. We examined whether mitochondrial elongation by itself contributes to the increase in mitochondrial ATP production in glucose-deprived cells. Treatment with siRNA specific for Mfn1, a protein mediating mitochondrial fusion, successfully blocked mitochondrial elongation in glucose-deprived cells, leaving fragmental mitochondria (Figure 7A). However, this treatment did not affect the increase in mitochondrial content, indicating that mitophagy still did not occur (Figure 7B). Moreover, the treatment marginally attenuated the increase in mitochondrial ATP production but did not suppress the change; thus, mitochondrial ATP production remained elevated albeit to slightly lower extents (Figure 7C,D). Further, the treatment decreased ATP level in glucose-fed cells too, together indicating that mitochondrial fragmentation did not hamper the increased production of ATP upon glucose withdrawal. Therefore, we suggest that the increased mitochondrial ATP synthesis is largely attributed to increased mitochondria biogenesis, independent of mitochondria structure.

## 4. Discussion

**Figure 8:**
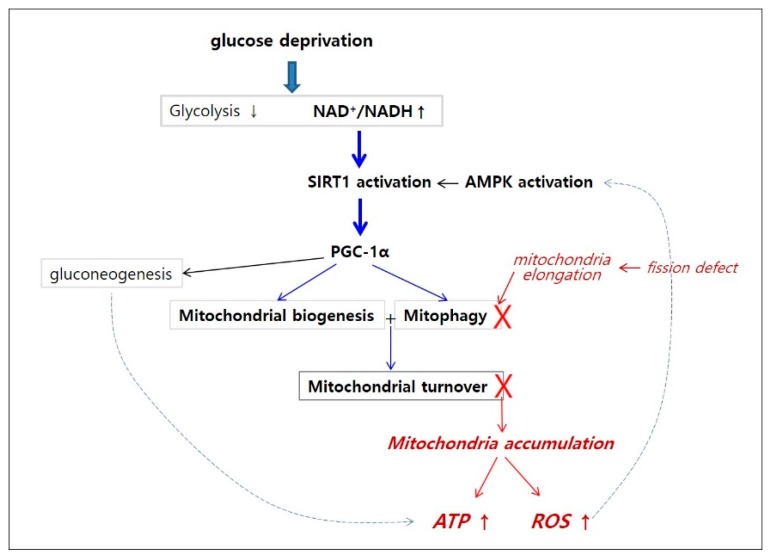
Hypothetical pathway for high-level production of ATP and ROS in glucose-deprived cells. Upon glucose deprivation, glycolysis level decreases causing an elevation in NAD+/NADH ratio in the cytosol, leading to SIRT1 activation. This activates PGC-1α, which triggers mitochondrial biogenesis and autophagy, and these together promote mitochondrial turnover (a pathway of SIRT1-mitochondrial quality control pathway (blue)). However, defects in mitochondria fission possibly caused by high-level ATP attenuate mitophagy, and induce the accumulation of mitochondria, from which high-level ATP and ROS are generated (a pathway of defective mitophagy (red)). PGC-1α also increases gluconeogenesis, promoting the increase of glycolysis, which further contributes to the elevation of cellular ATP level.

The results of our study showed that the cellular ATP level gradually increased under glucose withdrawal, despite an initial decrease in glycolysis, through a rapid increase in mitochondrial ATP production and the delayed recovery of glycolysis. This increased production of ATP was driven by an increase in mitochondrial content, which was attributed to an elevation of mitochondrial biogenesis mediated by SIRT1, which is activated by the increased ratio of NAD^+^/NADH. AMPK was activated, and it might function in conjunction with SIRT1 in mitochondria biogenesis. All of these changes were also seen in cancer cells under glucose withdrawal, suggesting that a decrease in glycolytic flux generally upregulates mitochondrial synthesis. SIRT1 activation normally enhances mitochondrial turnover through a balance between biogenesis and degradation, and enables cells to maintain a constant levels of ATP and ROS. This is key to cellular health, since a decrease in mitophagy as cells approach senescence constitutes a major reason for increased ROS and consequent induced oxidative damage [32]. In glucose-deprived cells, activated SIRT1 and AMPK did induce autophagy, but this was not extended to mitophagy, likely due to the dissociation with mitochondria fission. This imbalance in biogenesis and degradation of mitochondria constitutes the reason for the continuous increase in mitochondria content, and the accompanying elevation of ATP and ROS levels in glucose-deprived cells. This is summarized in Figure 8. (The process is hypothetically dissected into two pathways: a pathway of SIRT1-mitochondrial quality control and a pathway of defective mitophagy.) The involvement of SIRT1 in the concerted elevation of ATP and ROS levels in glucose-deprived cells is supported by the finding that the inhibition of SIRT1 caused a decrease in the level of both ATP and ROS, as well as mitochondria content. The correlation of ATP and ROS levels is also supported by the finding that levels of both increased upon feeding with 1.65 mM glucose, an equivalence of 30% of full feeding, while feeding with 3.3 mM glucose, corresponding to 60% of full feeding, did not cause a change in either (Figure S4). This indicates that the high level of ROS is an outcome of high-level ATP production, which largely results from an elevation in mitochondria content. A possible contribution of an increase in mitochondrial efficiency to the elevation of mitochondrial ATP production is also suggested in this study. The increase in mitochondrial ATP production and oxygen consumption exceeded the increase in mitochondria content, as shown in Figure S5A,B. These suggest that the increase in mitochondria biogenesis brings in the increase, not only of mitochondria quantity, but also of the efficiency of OXHOS, which might be facilitated through a SIRT1-mediated increase of biogenesis or activities of other sirtuin proteins [61].

The observation that glycolytic ATP production bounced back by 24 h can be explained by an increase in gluconeogenesis. Induction of PGC-1α-mediated gluconeogenesis by SIRT1 activation has been reported [46]; therefore, this is probably another outcome of SIRT1 activation. Interestingly, during the initial 24 h, the increase in glycolytic ATP synthesis accompanied a decrease in mitochondrial ATP synthesis as seen in Figure 1. In parallel to this, cellular oxygen consumption stopped increasing (or decreased) after 6 h point. Therefore, mitochondrial ATP synthesis may counterbalance glycolytic flux. There may exist a system where a change in the level of glycolysis is linked to the efficiency in OXPHOS. The nature of this system is unknown, but a delicate and transient shift in the balance of NAD^+^/NADH in cytosol, mitochondria, and nucleus may be involved. A study indicated that a decline in nuclear NAD^+^ level induces Hif-1α activation, causing a shift in ATP production parallel to Warburg reprogramming [62].

In previous studies, both increased and decreased ATP accumulation were reported in cancer and normal cells upon glucose withdrawal. This discrepancy may simply be due to the difference in the extent and duration of withdrawal. However, a literature search indicates that cases for the decrease in ATP level may be limited to results on murine primary cells, and are outnumbered by those on the increase found in human cells. For example, incubation in glucose-free medium caused a more than 20% increase in ATP levels in human aortic endothelial cells [63] and human normal astrocytes, as well as U251 MG glioblastoma multiform cells [13]. Different efficiencies of mitochondrial ATP synthesis in different tissues and animals may be involved. A recent study reported a variation in respiration/glycolysis ratio in ATP synthesis in sperm of different mouse strains [64]. Variations in mitochondrial efficiency affecting energy homeostasis in cells and tissues were also reported [65,66]. Meanwhile, an increase in ATP accumulation and a rapid shift from glycolysis to fatty acid oxidation and mitochondrial respiration have been reported in human aortic endothelial cells under glucose deprivation [63]. The study also proposed an involvement and a function of nicotinamide phosphoribosyltransferase (Nampt), a critical enzyme in the salvage production of NAD^+^ [22], in the cellular response to glucose level, and thereby critical modulation. Therefore, the status of SIRT1 and the NAD^+^ pool may also differ in different cells and tissues, and determine the level of mitochondrial ATP synthesis upon glucose withdrawal. In our study, glucose withdrawal differently affected the fates of normal and cancer cells. The increased NAD^+^/NADH ratio would affect the level of anabolism in glucose-deprived cells. An effect of high-level glycolysis with limited OXPHOS, a condition found in cancer cells [38], is the maintenance of high levels of reducing power for macromolecular synthesis [67]. In glucose-free medium, normal fibroblasts proliferated at a normal rate for 24 h, but then stopped increasing in number without apparent death during the three days of testing (Figure S1E). The cells were unable to multiply, even in the presence of a large amount of ATP, likely due to a shortage in reducing power. In contrast, cancer cells, especially MCF7 cells, died rapidly, and their viability curve resembled the change in ATP level (Figure 1F, Figure S6B). Together, these findings suggest that the depletion of ATP directly affects cell viability, while the depletion of reducing power attenuates cell proliferation. Indeed, this effect has been conceived as an underlying mechanism of selective death of cancer cells by glucose depletion, and was proposed as an anti-cancer strategy [68,69]. It was also proposed that the susceptibility of cancer cells to glucose deprivation is attributed to the mitochondrial production of ROS [12]. A high level of ROS and a low level of ATP together would easily drive cells to death.

Another notable finding of our study is that mitochondria elongated upon glucose withdrawal. Mitochondrial elongation has been noted previously, and was proposed to be advantageous in nutrient restriction conditions by facilitating the better preservation of crista and ATP synthesis [70]. It would also be a good strategy for mitochondria to escape autophagosomal degradation. In the total absence of nutrients, the cAMP level increased, together with PKA activity, leading to the phosphorylation and inactivation of the pro-fission factor, Drp1 [58]. SIRT1 activation is known to induce mitochondria fragmentation [35]. The fact that mitochondria elongation occurred in the presence of active SIRT1 suggests that Drp1 phosphorylation in glucose withdrawal overcomes SIRT1 activity. Another question regarding mitochondrial elongation is whether it occurs as an effect to protect mitochondria from sustained autophagy activation, or in response to a need for a higher level of mitochondrial ATP production, as proposed by Gomes et al. [58]. The latter seems to be less plausible, since the results of our study showed that mitochondria were elongated, even when the ATP level was already high, in glucose-deprived cells. In the former case, mitochondria would become shortened once autophagy returns to basal level; indeed, mitochondrial shape returned to normal upon replenishment of glucose for 12 h (Figure S3B). This has also been observed in other starvation conditions (S.B. Song, data not shown). Therefore, mitochondrial elongation is more likely a means to avoid autophagy.

Overall, our study demonstrated that the cellular response to glucose withdrawal is mediated mainly through SIRT1 activation. However, we also showed that the downstream events of SIRT1 activation might not always proceed in coordination. Other such examples might be found in further studies on abnormal energy metabolism. Our results also shed light on events that might take place in cells upon extreme calorie restriction or fasting. In fasting, mitochondrial ATP production is mediated largely by the increased β-oxidation of fatty acids [71] and involves the activation of SIRT1 and SIRT3 [34,71]. Whether the mitochondria change shape during fasting warrants further examination.

## Supplementary Materials

The following are available online at http://www.mdpi.com/2073-4409/8/1/11/s1, Figure S1: Changes in total ATP level and mitochondrial ATP production and cell proliferation rates in different fibroblasts lines, Figure S2: Changes in the levels of phosphor AMPK, superoxides, and mitochondria content in HCT116 or MCF7 cells, Figure S3: Mitochondrial elongation upon glucose withdrawal, Figure S4: Effects of lowered glucose supply on the levels of mitochondria content and ROS, Figure S5: Changes in mitochondrial ATP production and oxygen consumption per unit mass of mitochondria, Figure S6: Effect of glucose withdrawal on cancer cell proliferation, Figure S7: Sequences of primers used for quantitative PCR.

## References

[1] Hardie D.G. (2011). Amp-activated protein kinase: An energy sensor that regulates all aspects of cell function. Genes Dev..

[2] Oakhill J.S., Steel R., Chen Z.P., Scott J.W., Ling N., Tam S., Kemp B.E. (2011). AMPK is a direct adenylate charge-regulated protein kinase. Science.

[3] Inoki K., Ouyang H., Zhu T., Lindvall C., Wang Y., Zhang X., Yang Q., Bennett C., Harada Y., Stankunas K. (2006). TSC2 integrates wnt and energy signals via a coordinated phosphorylation by AMPK and GSK3 to regulate cell growth. Cell.

[4] Jeon S.M. (2016). Regulation and function of AMPK in physiology and diseases. Exp. Mol. Med..

[5] Zong H., Ren J.M., Young L.H., Pypaert M., Mu J., Birnbaum M.J., Shulman G.I. (2002). AMP kinase is required for mitochondrial biogenesis in skeletal muscle in response to chronic energy deprivation. Proc. Natl. Acad. Sci. USA.

[6] Moley K.H., Mueckler M.M. (2000). Glucose transport and apoptosis. Apoptosis.

[7] Demetrakopoulos G.E., Linn B., Amos H. (1982). Starvation, deoxy-sugars, ouabain, and ATP metabolism in normal and malignant cells. Cancer Biochem. Biophys..

[8] Xu R.H., Pelicano H., Zhou Y., Carew J.S., Feng L., Bhalla K.N., Keating M.J., Huang P. (2005). Inhibition of glycolysis in cancer cells: A novel strategy to overcome drug resistance associated with mitochondrial respiratory defect and hypoxia. Cancer Res..

[9] Buzzai M., Bauer D.E., Jones R.G., Deberardinis R.J., Hatzivassiliou G., Elstrom R.L., Thompson C.B. (2005). The glucose dependence of Akt-transformed cells can be reversed by pharmacologic activation of fatty acid beta-oxidation. Oncogene.

[10] Blackburn R.V., Spitz D.R., Liu X., Galoforo S.S., Sim J.E., Ridnour L.A., Chen J.C., Davis B.H., Corry P.M., Lee Y.J. (1999). Metabolic oxidative stress activates signal transduction and gene expression during glucose deprivation in human tumor cells. Free Radic. Biol. Med..

[11] Lee Y.J., Galoforo S.S., Berns C.M., Chen J.C., Davis B.H., Sim J.E., Corry P.M., Spitz D.R. (1998). Glucose deprivation-induced cytotoxicity and alterations in mitogen-activated protein kinase activation are mediated by oxidative stress in multidrug-resistant human breast carcinoma cells. J. Biol. Chem..

[12] Ahmad I.M., Aykin-Burns N., Sim J.E., Walsh S.A., Higashikubo R., Buettner G.R., Venkataraman S., Mackey M.A., Flanagan S.W., Oberley L.W. (2005). Mitochondrial O2*- and H2O2 mediate glucose deprivation-induced stress in human cancer cells. J. Biol. Chem..

[13] Jelluma N., Yang X., Stokoe D., Evan G.I., Dansen T.B., Haas-Kogan D.A. (2006). Glucose withdrawal induces oxidative stress followed by apoptosis in glioblastoma cells but not in normal human astrocytes. Mol. Cancer Res..

[14] Liu Y., Song X.D., Liu W., Zhang T.Y., Zuo J. (2003). Glucose deprivation induces mitochondrial dysfunction and oxidative stress in PC12 cell line. J. Cell. Mol. Med..

[15] Williams T., Forsberg L.J., Viollet B., Brenman J.E. (2009). Basal autophagy induction without AMP-activated protein kinase under low glucose conditions. Autophagy.

[16] Marambio P., Toro B., Sanhueza C., Troncoso R., Parra V., Verdejo H., Garcia L., Quiroga C., Munafo D., Diaz-Elizondo J. (2010). Glucose deprivation causes oxidative stress and stimulates aggresome formation and autophagy in cultured cardiac myocytes. Biochim. Biophys. Acta.

[17] Salt I.P., Johnson G., Ashcroft S.J., Hardie D.G. (1998). AMP-activated protein kinase is activated by low glucose in cell lines derived from pancreatic beta cells, and may regulate insulin release. Biochem. J..

[18] Healy D.A., Watson R.W., Newsholme P. (2002). Glucose, but not glutamine, protects against spontaneous and anti-fas antibody-induced apoptosis in human neutrophils. Clin. Sci..

[19] Moore C.E., Omikorede O., Gomez E., Willars G.B., Herbert T.P. (2011). PERK activation at low glucose concentration is mediated by SERCA pump inhibition and confers preemptive cytoprotection to pancreatic beta-cells. Mol. Endocrinol..

[20] Sakurai T., Yang B., Takata T., Yokono K. (2002). Synaptic adaptation to repeated hypoglycemia depends on the utilization of monocarboxylates in guinea pig hippocampal slices. Diabetes.

[21] Hsu C.P., Oka S., Shao D., Hariharan N., Sadoshima J. (2009). Nicotinamide phosphoribosyltransferase regulates cell survival through NAD+ synthesis in cardiac myocytes. Circ. Res..

[22] Fulco M., Cen Y., Zhao P., Hoffman E.P., McBurney M.W., Sauve A.A., Sartorelli V. (2008). Glucose restriction inhibits skeletal myoblast differentiation by activating sirt1 through AMPK-mediated regulation of nampt. Dev. Cell..

[23] Gold A.J., Yaffe S.R. (1978). Effects of prolonged starvation on cardiac energy metabolism in the rat. J. Nutr..

[24] Zmijewski J.W., Banerjee S., Bae H., Friggeri A., Lazarowski E.R., Abraham E. (2010). Exposure to hydrogen peroxide induces oxidation and activation of AMP-activated protein kinase. J. Biol. Chem..

[25] Cardaci S., Filomeni G., Ciriolo M.R. (2012). Redox implications of AMPK-mediated signal transduction beyond energetic clues. J. Cell. Sci..

[26] Swerdlow R.H. (2009). Mitochondrial medicine and the neurodegenerative mitochondriopathies. Pharmaceuticals.

[27] Chertov A.O., Holzhausen L., Kuok I.T., Couron D., Parker E., Linton J.D., Sadilek M., Sweet I.R., Hurley J.B. (2011). Roles of glucose in photoreceptor survival. J. Biol. Chem..

[28] Bordone L., Guarente L. (2005). Calorie restriction, sirt1 and metabolism: Understanding longevity. Nat. Rev. Mol. Cell. Biol..

[29] Canto C., Auwerx J. (2009). Caloric restriction, SIRT1 and longevity. Trends Endocrinol. Metab..

[30] Lee I.H., Cao L., Mostoslavsky R., Lombard D.B., Liu J., Bruns N.E., Tsokos M., Alt F.W., Finkel T. (2008). A role for the NAD-dependent deacetylase Sirt1 in the regulation of autophagy. Proc. Natl. Acad. Sci. USA.

[31] Huang R., Xu Y., Wan W., Shou X., Qian J., You Z., Liu B., Chang C., Zhou T., Lippincott-Schwartz J. (2015). Deacetylation of nuclear LC3 drives autophagy initiation under starvation. Mol. Cell..

[32] Guarente L. (2008). Mitochondria–a nexus for aging, calorie restriction, and sirtuins?. Cell.

[33] Jendrach M., Pohl S., Voth M., Kowald A., Hammerstein P., Bereiter-Hahn J. (2005). Morpho-dynamic changes of mitochondria during ageing of human endothelial cells. Mech. Ageing Dev..

[34] Picard F., Kurtev M., Chung N., Topark-Ngarm A., Senawong T., Machado De Oliveira R., Leid M., McBurney M.W., Guarente L. (2004). Sirt1 promotes fat mobilization in white adipocytes by repressing PPAR-gamma. Nature.

[35] Jang S.Y., Kang H.T., Hwang E.S. (2012). Nicotinamide-induced mitophagy: Event mediated by high NAD+/NADH ratio and SIRT1 protein activation. J. Biol. Chem..

[36] Zhang Y., Marsboom G., Toth P.T., Rehman J. (2013). Mitochondrial respiration regulates adipogenic differentiation of human mesenchymal stem cells. PLoS ONE.

[37] Tarrado-Castellarnau M., de Atauri P., Tarrago-Celada J., Perarnau J., Yuneva M., Thomson T.M., Cascante M. (2017). De novo MYC addiction as an adaptive response of cancer cells to CDK4/6 inhibition. Mol. Syst Biol.

[38] Warburg O. (1956). On the origin of cancer cells. Science.

[39] Chen X., Qian Y., Wu S. (2015). The Warburg effect: Evolving interpretations of an established concept. Free Radic. Biol. Med..

[40] Wang Q., Liang B., Shirwany N.A., Zou M.H. (2011). 2-deoxy-d-glucose treatment of endothelial cells induces autophagy by reactive oxygen species-mediated activation of the AMP-activated protein kinase. PLoS ONE.

[41] Vaziri H., Dessain S.K., Ng Eaton E., Imai S.I., Frye R.A., Pandita T.K., Guarente L., Weinberg R.A. (2001). Hsir2(sirt1) functions as an NAD-dependent p53 deacetylase. Cell.

[42] Chu C.T. (2010). A pivotal role for PINK1 and autophagy in mitochondrial quality control: Implications for Parkinson disease. Hum. Mol. Genet..

[43] Hwang E.S., Song S.B. (2017). Nicotinamide is an inhibitor of sirt1 in vitro, but can be a stimulator in cells. Cell. Mol. Life Sci..

[44] Wu Z., Puigserver P., Andersson U., Zhang C., Adelmant G., Mootha V., Troy A., Cinti S., Lowell B., Scarpulla R.C. (1999). Mechanisms controlling mitochondrial biogenesis and respiration through the thermogenic coactivator PGC-1. Cell.

[45] Nemoto S., Fergusson M.M., Finkel T. (2005). SIRT1 functionally interacts with the metabolic regulator and transcriptional coactivator PGC-1{alpha}. J. Biol. Chem..

[46] Rodgers J.T., Lerin C., Haas W., Gygi S.P., Spiegelman B.M., Puigserver P. (2005). Nutrient control of glucose homeostasis through a complex of PGC-1alpha and SIRT1. Nature.

[47] Liang J., Shao S.H., Xu Z.X., Hennessy B., Ding Z., Larrea M., Kondo S., Dumont D.J., Gutterman J.U., Walker C.L. (2007). The energy sensing LKB1-AMPK pathway regulates p27(kip1) phosphorylation mediating the decision to enter autophagy or apoptosis. Nat. Cell. Biol..

[48] Meley D., Bauvy C., Houben-Weerts J.H., Dubbelhuis P.F., Helmond M.T., Codogno P., Meijer A.J. (2006). AMP-activated protein kinase and the regulation of autophagic proteolysis. J. Biol. Chem..

[49] Law B.Y., Wang M., Ma D.L., Al-Mousa F., Michelangeli F., Cheng S.H., Ng M.H., To K.F., Mok A.Y., Ko R.Y. (2010). Alisol b, a novel inhibitor of the sarcoplasmic/endoplasmic reticulum Ca(2+) ATPase pump, induces autophagy, endoplasmic reticulum stress, and apoptosis. Mol. Cancer Ther..

[50] Yoon J.C., Puigserver P., Chen G., Donovan J., Wu Z., Rhee J., Adelmant G., Stafford J., Kahn C.R., Granner D.K. (2001). Control of hepatic gluconeogenesis through the transcriptional coactivator PGC-1. Nature.

[51] Behrends C., Sowa M.E., Gygi S.P., Harper J.W. (2010). Network organization of the human autophagy system. Nature.

[52] Ding W.X., Yin X.M. (2012). Mitophagy: Mechanisms, pathophysiological roles, and analysis. Biol. Chem..

[53] Twig G., Elorza A., Molina A.J., Mohamed H., Wikstrom J.D., Walzer G., Stiles L., Haigh S.E., Katz S., Las G. (2008). Fission and selective fusion govern mitochondrial segregation and elimination by autophagy. EMBO J..

[54] Rambold A.S., Kostelecky B., Elia N., Lippincott-Schwartz J. (2011). Tubular network formation protects mitochondria from autophagosomal degradation during nutrient starvation. Proc. Natl. Acad. Sci. USA.

[55] Chang C.R., Blackstone C. (2007). Cyclic AMP-dependent protein kinase phosphorylation of Drp1 regulates its GTPase activity and mitochondrial morphology. J. Biol. Chem..

[56] Cribbs J.T., Strack S. (2007). Reversible phosphorylation of Drp1 by cyclic AMP-dependent protein kinase and calcineurin regulates mitochondrial fission and cell death. EMBO Rep..

[57] Dickey A.S., Strack S. (2011). PKA/AKAP1 and PP2A/Bβ2 regulate neuronal morphogenesis via Drp1 phosphorylation and mitochondrial bioenergetics. J. Neurosci..

[58] Gomes L.C., Di Benedetto G., Scorrano L. (2011). During autophagy mitochondria elongate, are spared from degradation and sustain cell viability. Nat. Cell. Biol..

[59] Mitra K., Wunder C., Roysam B., Lin G., Lippincott-Schwartz J. (2009). A hyperfused mitochondrial state achieved at G1-S regulates cyclin e buildup and entry into s phase. Proc. Natl Acad. Sci. USA.

[60] Rolland S.G., Motori E., Memar N., Hench J., Frank S., Winklhofer K.F., Conradt B. (2013). Impaired complex iv activity in response to loss of LRPPRC function can be compensated by mitochondrial hyperfusion. Proc. Natl. Acad. Sci. USA.

[61] North B.J., Sinclair D.A. (2007). Sirtuins: A conserved key unlocking acecs activity. Trends Biochem. Sci..

[62] Gomes A.P., Price N.L., Ling A.J., Moslehi J.J., Montgomery M.K., Rajman L., White J.P., Teodoro J.S., Wrann C.D., Hubbard B.P. (2013). Declining NAD(+) induces a pseudohypoxic state disrupting nuclear-mitochondrial communication during aging. Cell.

[63] Borradaile N.M., Pickering J.G. (2009). Nicotinamide phosphoribosyltransferase imparts human endothelial cells with extended replicative lifespan and enhanced angiogenic capacity in a high glucose environment. Aging Cell..

[64] Tourmente M., Roldan E.R. (2015). Mass-specific metabolic rate influences sperm performance through energy production in mammals. PLoS ONE.

[65] Pham A.H., Chan D.C. (2014). Analyzing mitochondrial dynamics in mouse organotypic slice cultures. Methods Enzymol..

[66] Layec G., Bringard A., Le Fur Y., Micallef J.P., Vilmen C., Perrey S., Cozzone P.J., Bendahan D. (2015). Opposite effects of hyperoxia on mitochondrial and contractile efficiency in human quadriceps muscles. Am. J. Physiol. Regul. Integr. Comp. Physiol..

[67] Brand K.A., Hermfisse U. (1997). Aerobic glycolysis by proliferating cells: A protective strategy against reactive oxygen species. FASEB J..

[68] Ganapathy-Kanniappan S., Geschwind J.F. (2013). Tumor glycolysis as a target for cancer therapy: Progress and prospects. Mol. Cancer..

[69] Wei S., Kulp S.K., Chen C.S. (2010). Energy restriction as an antitumor target of thiazolidinediones. J. Biol. Chem..

[70] Gomes L.C., Scorrano L. (2011). Mitochondrial elongation during autophagy: A stereotypical response to survive in difficult times. Autophagy.

[71] Hirschey M.D., Shimazu T., Goetzman E., Jing E., Schwer B., Lombard D.B., Grueter C.A., Harris C., Biddinger S., Ilkayeva O.R. (2010). Sirt3 regulates mitochondrial fatty-acid oxidation by reversible enzyme deacetylation. Nature.

