# Supplementary material for: A Rise in ATP, ROS, and Mitochondrial Content upon Glucose Withdrawal Correlates with a Dysregulated Mitochondria Turnover Mediated by the Activation of the Protein Deacetylase SIRT1
Source: Cells. 2018 Dec 27;8(1):11. doi: 10.3390/cells8010011 (PMC6356350; doi:10.3390/cells8010011)
Supplement: Supplementary file 1 [file cells-08-00011-s001.pdf]

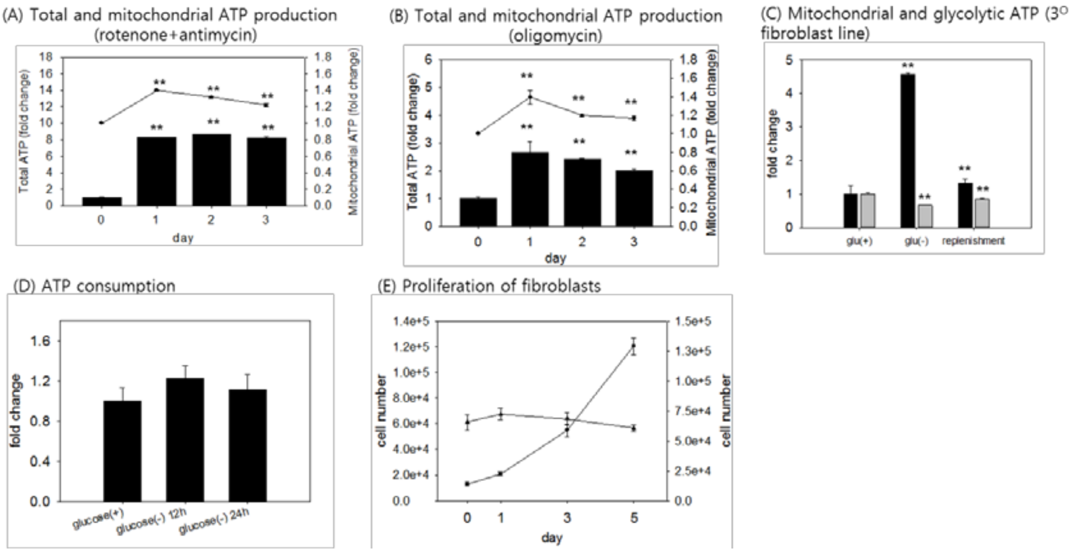

Figure S1. Changes in total ATP level and mitochondrial ATP production and cell proliferation rates in different fibroblasts lines; (**A**) and (**B**) changes in total ATP level (line plot) and mitochondrial ATP production (bar chart) in fibroblasts of the second donor incubated in glucose-free medium for the indicated times. Mitochondrial ATP production was calculated by subtraction of glycolytic ATP from total ATP. For measurement of glycolytic ATP production, oxidative phosphorylation (OXPHOS) was inhibited by combined treatment with 1 μM antimycin A and 1 μM rotenone for the last hour of glucose deprivation (**A**) or 2 μM oligomycin for the last 2 hours (**B**); (**C**) changes in the levels of mitochondrial (black bar) and glycolytic (grey bar) ATP production in fibroblasts of the third donor. The effect of glucose replenishment on ATP level was also determined by cultivating cells in glucose-free medium for 3 days and then re-feeding glucose for the last 1 day. Relative changes in ATP synthesis from OXPHOS (black bar) and from glycolysis (grey bar) were plotted. (**D**) ATP consumption rates in glucose-fed and -deprived cells. Cellular ATP levels in the cells treated with 10 mM 2-DG and 2 μM oligomycin A for 2 hr at 12 h or 24 h glucose withdrawal (black bars) are not much different with those in the cells treated in the presence of glucose (grey bars). (**E**) Changes in cell proliferation rate. Fibroblasts (of the first donor) were incubated in normal (-•-) or glucose-free (-▲-) medium for 5 days, and the number of cells was counted at the indicated time points. Values are presented as mean ± s.d. *$p < 0.05$; **$p < 0.01$ by ANOVA.

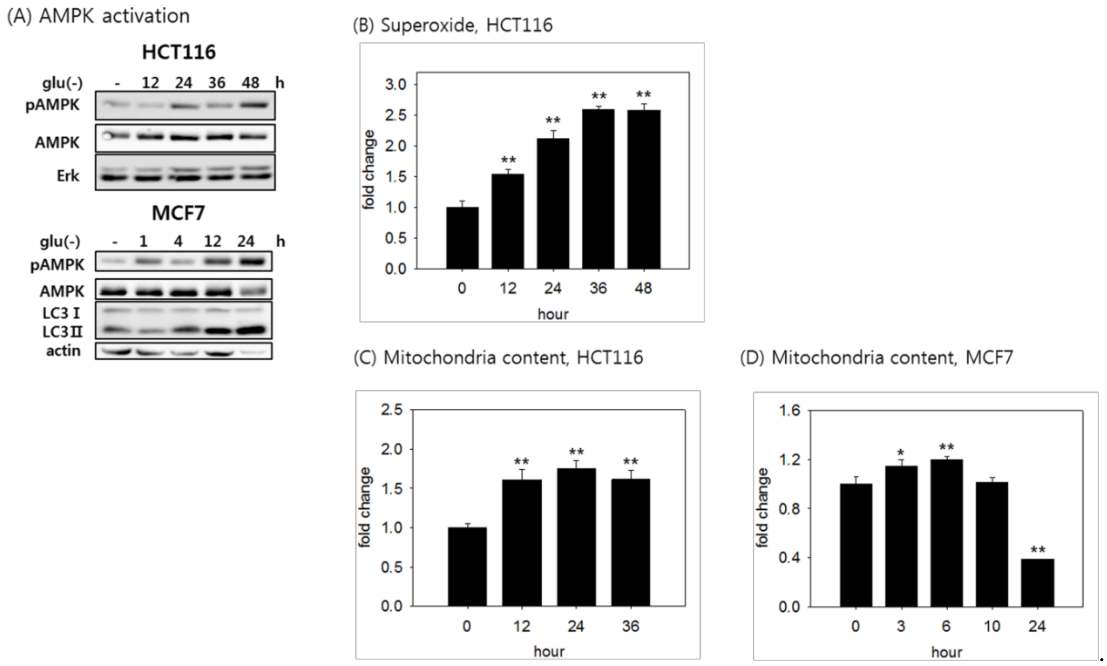

Figure S2. Changes in the levels of phospho AMPK, superoxides, and mitochondria content in HCT116 or MCF7 cells; (**A**) HCT116 or MCF7 cells were incubated in glucose-free medium for the indicated time period and harvested. Activity of AMPK was detected by immunoblotting with antibodies against phospho-AMPK and AMPK; (**B**) HCT116 cells were glucose-starved for 48 h, stained with mitoSOX, and analyzed by flow cytometry; (**C**, **D**) Mitochondrial content in HCT116 or MCF7 cells was measured through flow cytometric analysis of staining with MTG or NAO. Values are presented as mean ± s.d.; $^*p < 0.05$, $^{**}p < 0.01$ by ANOVA.

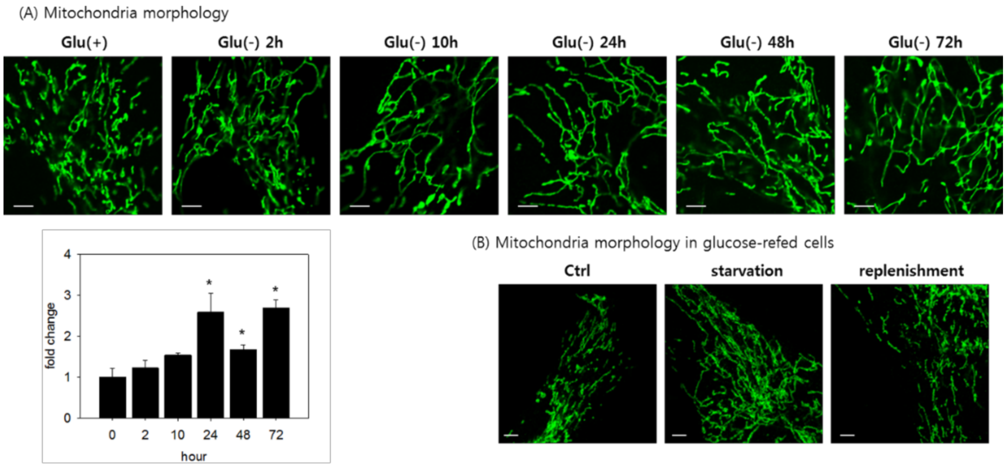

Figure S3. Mitochondrial elongation upon glucose withdrawal; (**A**) fibroblasts cultivated on cover slips were glucose deprived for indicated duration and immunostained with OXPHOS antibody. Morphology of mitochondria was visualized by confocal microscopy. The length of mitochondria was measured by ImageJ software and graphed below; (**B**) Fibroblasts were cultivated in glucose-free medium for 3 days and then re-fed with glucose for an additional 12 h. Mitochondria were

immunostained with antibody against Tom20. All scale bars in microscopy indicate 5 μm. Values are presented as mean ± s.d.; *$p < 0.05$, **$p < 0.01$ by ANOVA.

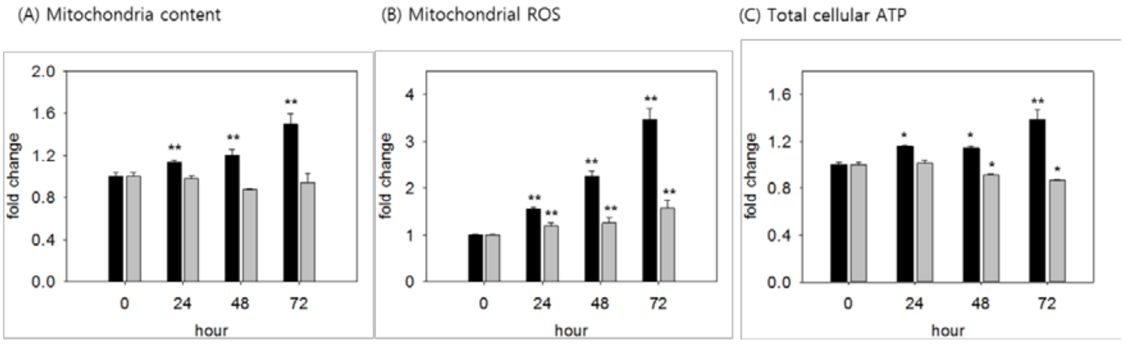

Figure S4. Effects of lowered glucose supply on the levels of mitochondria content and ROS; (**A**, **B**) fibroblasts were cultivated in medium containing 30% (black bar) or 60% (grey bar) of normal glucose concentration. After incubation for 3 days, cells were stained with NAO or mitoSOX and subjected to flow cytometry for detection of mitochondrial content (**A**) or mitochondrial superoxide (**B**), respectively; (**C**) Total cellular ATP level was measured in cells incubated in medium containing 30% (black bar) or 60% (grey bar) glucose for 3 days. Values are presented as mean ± s.d.; *$p < 0.05$, **$p < 0.01$ by ANOVA.

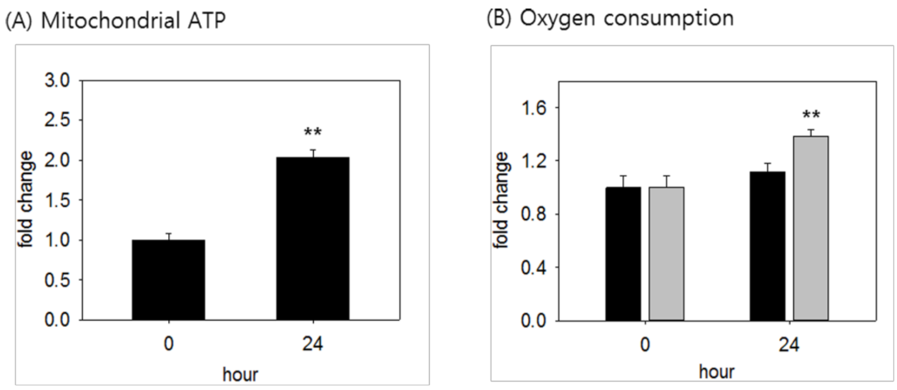

Figure S5. Changes in mitochondrial ATP production and oxygen consumption per unit mass of mitochondria. Mitochondrial ATP production (determined by using MTG) (**A**) and oxygen consumption (**B**) determined in Figures 1B and 1C at 0 h and 24 h of glucose deprivation were divided by the mitochondrial content determined in Figure 4A at the same time point. The numbers relative to that of 0 h is plotted. Values are presented as mean ± s.d.; *$p < 0.05$, **$p < 0.01$ by ANOVA.

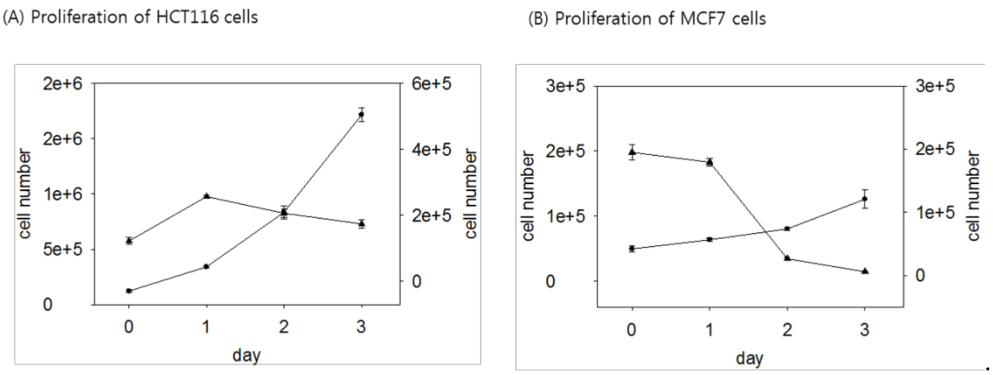

Figure S6. Effect of glucose withdrawal on cancer cell proliferation; (**A**, **B**) HCT116 or MCF7 cells were incubated in normal (-●-) or glucose-free (-▲-) medium for 3 days, and the number of cells was counted at the indicated time points.

ND5, 5′-CAGCAGCCATTCAAGCAATGC-3′ and 5′-GGTGGAGACCTAATTGGGCTG ATTAG-3′;
ND6, 5′-ATAGGATCCTCCCGAATCAACCCT-3′ and 5'-AGGATTGGTGCTGTGGGTGAAAGA-3′;
MT-COX2, 5′-ACAGATGCAATTCCCGGACGTCTA-3′ and 5′-GGCATGAAACTGTGGTTTGCTCCA-3′;
Atp5g1, 5′-TGGCAGCTTGATCATTGGCTATGC-3′ and 5′-AGGATGAGGAAGGCGACCATCAAA-3′;
Cyt C, 5′-TGGGCCAAATCTCCATGGTCTCTT-3′ and 5′-TGCCTTTGTTCTTATTGGCGGCTG-3′;
PGC-1a, 5′-GGCAGTAGATCCTCTTCAAGATC-3′ and 5′-TCACACGGCGCTCTTCAATTG-3′;
TFAM, 5′-AACCCTGATGGCACTGTCTCACTT-3′ and 5′-TCTCCATCAGCCACGGCAGAATAA-3′;
NRF1, 5′-AGGCACAGGAAACCAGTTAGGTCT-3′ and 5′-TTATGCTGGCAGAAGTCCATGAGC-3′.
tRNA-Leu: 5′-CACCCAAGAACAGGGTTTGT-3′ and 5′-TGGCCATGGGTATGTTGTTA-3′
nuclear B2-microglobulin: 5′-TGCTGTCTCCATGTTTGATGTATCT-3′ and 5′-TCTCTGCTCCCCACCTCTAAGT-3′.

Figure S7. Sequences of primers used for quantitative PCR.
